# Building an Artificial Stem Cell Niche: Prerequisites for Future 3D‐Formation of Inner Ear Structures—Toward 3D Inner Ear Biotechnology

**DOI:** 10.1002/ar.24067

**Published:** 2019-01-28

**Authors:** Simon C. de Groot, Karen Sliedregt, Peter Paul G. van Benthem, Marcelo N. Rivolta, Margriet A. Huisman

**Affiliations:** ^1^ Hair Science Institute, Maastricht Maastricht the Netherlands; ^2^ Wageningen University and Research Wageningen the Netherlands; ^3^ Department of Otorhinolaryngology and Head & Neck Surgery Leiden University Medical Center Leiden the Netherlands; ^4^ Centre for Stem Cell Biology, Department of Biomedical Science University of Sheffield Sheffield UK

**Keywords:** inner ear, 3D, stem cell, biotechnology

## Abstract

In recent years, there has been an increased interest in stem cells for the purpose of regenerative medicine to deliver a wide range of therapies to treat many diseases. However, two‐dimensional cultures of stem cells are of limited use when studying the mechanism of pathogenesis of diseases and the feasibility of a treatment. Therefore, research is focusing on the strengths of stem cells in the three‐dimensional (3D) structures mimicking organs, that is, organoids, or organ‐on‐chip, for modeling human biology and disease. As 3D technology advances, it is necessary to know which signals stem cells need to multiply and differentiate into complex structures. This holds especially true for the complex 3D structure of the inner ear. Recent work suggests that although other factors play a role, the extracellular matrix (ECM), including its topography, is crucial to mimic a stem cell niche *in vitro* and to drive stem cells toward the formation of the tissue of interest. Technological developments have led to the investigation of biomaterials that closely resemble the native ECM. In the fast forward moving research of organoids and organs‐on‐chip, the inner ear has hardly received attention. This review aims to provide an overview, by describing the general context in which cells, matrix and morphogens cooperate in order to build a tissue, to facilitate research in 3D inner ear technology. Anat Rec, 303:408–426, 2020. © 2019 The Authors. *The Anatomical Record* published by Wiley Periodicals, Inc. on behalf of American Association of Anatomists.

Stem cells differ from other cells in the body. In general, stem cells have a few unique characteristics over somatic cells; they are undifferentiated and can give rise to differentiated daughter cells and some stem cells have the ability to divide and renew above the Hayflick limit of somatic cells (Ding and Schultz, 2004; Young et al., 2004; Armstrong and Tomita, 2017). These abilities are of interest in the field of regenerative medicine to repair or renew damaged tissue (Thomson et al., 1998; Watt and Driskell, 2010; Whiting et al., 2015). Stem cells can be categorized into three main groups: embryonic stem cells (ESCs), induced pluripotent stem cells (iPSCs), and adult stem cells. Literature defines an ESC and iPSC as pluripotent, that is, having the capacity to form all three germ layers: endoderm, mesoderm, and ectoderm (Griffin et al., 2015). IPSCs can be generated from mouse and human somatic cells by artificially overexpressing four genes (Oct3‐4, Sox2, c‐Myc, and Klf4 or OCT4, SOX2, NANOG, and LIN28 (Takahashi and Yamanaka, 2006; Yu et al., 2007). Due to their pluripotency and the ability to self‐renew, ESCs and iPSC are very suitable candidates for regenerative medicine. However, controlling differentiation and proliferation *in vitro* appeared to be challenging (Levenberg et al., 2003). Moreover, as a consequence of their highly proliferative nature, undifferentiated ESCs and iPSCs are prone to develop into cancer cells, for example, into teratoma *in vivo*, limiting their use in clinical trials (Hentze et al., 2009; Knoepfler, 2009). Culture model systems, generated from iPSCs from patients with genetic mutations, can be used to study the onset of disease or to explore pharmacological interventions (Qian et al., 2016).

The last group of stem cells, adult stem cells, resides in various tissues within the body, typically at places with a high cell turnover rate such as the gut, skin, and blood (Wagers and Weissman, 2004). In general, adult stem cells are multipotent, that is, they can only differentiate within one germ layer. Mesenchymal stem cells (MSCs) for example, are capable to differentiate toward most cells derived from the mesodermal germ layer such as bone marrow stroma, adipose tissue, osteocytes, and chondrocytes (Abdallah and Kassem, 2008). In addition, multipotent stem cells have a lower proliferation rate than pluripotent stem cells and hence adult stem cells are not tumor‐prone. For the purpose of “personalized medicine,” adult stem cells have the advantage that they can be used for autologous cell‐based therapies. Due to the restricted potency of adult stem cells, their capacity to grow into organoids is limited. Nevertheless, promising results have been achieved with culturing epithelial organoid cultures from human intestinal crypts (Dotti et al., 2017). It has been shown that many stem cell types are capable to differentiate *in vitro* into the desired cell lineage with the right combination of stimulatory and inhibitory factors. However, in two‐dimensional (2D) cultures, their ability to form stable, functional cell types and complicated structures are very limited (Kaufman et al., 2001; Reubinoff et al., 2001; Levenberg et al., 2003). A potentially important issue is the difference in oxygen consumption. In 2D cultures, all cells are exposed to the same oxygen tension, that is, their oxygen consumption rates are constant. This contrasts with cells in three‐dimensional (3D)‐culture whereas oxygen diffuses into the complex structure and some cells “see” less oxygen and average consumption per cell is lower approaching rates of consumption measured *in vivo* (Streeter and Cheema, 2011). However, organoids should not grow too much in size, for nutrition and oxygen supply throughout the whole tissue becomes more challenging because organoids often lack vascularization. Areas with poor oxygen supply and nutrition often lead to differentiation of cells into an undesired cell type and limit maturation of the organoid (Chambers et al., 2013).

In 3D‐cultures, it is key to bio‐engineer the right scaffold to study cellular mechanics which drive (stem) cell fate and to study the role of the stem cell niche. The natural microenvironment of cells *in vivo* consists of an extracellular matrix (ECM) which contains a mixture of proteins arranged into complex topographic features that guide cells toward their phenotype (McNamara et al., 2010). Aside from cell specialization, the (3D) ECM is involved in many aspects in the life of cells, such as cell adhesion, proliferation, migration, and suppression of inhibitory signals (Daley et al., 2008). In this perspective, it is believed that not only biochemical but also biophysical cues such as stiffness and topography of the ECM, together with direct cell–cell contact are of great importance in controlling stem cell fate (Yao et al., 2013; Lv et al., 2015). Increasing evidence supports that 3D culture in pertinent scaffolds is not only necessary to control stem cell proliferation and differentiation but that it is also crucial in the development of stem cells into higher order structures such as organoids (Langer and Vacanti, 1993; Atala, 2012). The combination of organoid and stem cell technology is a promising concept in both developmental and regenerative research. Importantly, the culture of organoids helps to establish specific morphogen gradients, which are required for the generation of tissue organoids of a particular identity (Akkerman and Defize, 2017). Culturing cells in a 3D matrix enhance their expression of differentiated functions and improve their organization but fail to reconstitute (parts of) living organs. Another drawback for usage of organoids in 3D culture is that organoids can vary a lot in size and shape and those cells deep in the organoid are hard to visualize, even with high‐resolution imaging (Bhatia and Ingber, 2014). Moreover, mimicking complicated *in vivo* processes such as physiological diffusion gradients (e.g., ion transport) is not possible. It is for these reasons that cell and disease studies remain largely dependent on time‐consuming and costly animal studies, although these in many cases failed to predict human responses (Huh et al., 2011). Organ‐on‐chip technology may present solutions to these challenges and the next generation of 3D cell culture models are developed, to better mimic the microstructure, dynamic mechanical properties, and biochemical functionalities of whole living organs. These organs‐on‐chips contain multiple micro‐sized chips designed to simulate the physiological conditions of tissues and organs (Bhatia and Ingber, 2014). These microfluidic devices allow precise control of cells, fluids, and oxygen at the nanoliter scale and facilitate simultaneous manipulation and analysis of cultured cells. Hence, the possibility to control fluid flow in a microfluidic device enhances differentiation and survival of various cell types, including neural stem cells (Cimetta et al., 2010; Huh et al., 2010). The use of microfluidic cell culture devices can give a great advance to study tissue development, diseases, and organ physiology (Bhatia and Ingber, 2014). A valuable application of the organ‐on‐chip model has recently been demonstrated by Wang et al. (2014) who compared patient‐derived and disease‐specific genetically engineered iPCS. Using the organ‐on‐chip technique, they were able to validate the causal effect of the genetic mutation. Another approach to tissue engineering is cell sheet engineering (Auger et al., 2000). Temperature‐responsive polymers are covalently bound on culture dishes which allows adhesion and culture at 37°C and subsequent detachment of cells. The sheet, formed by the confluent cells spontaneously detaches when the temperature is reduced below 32°C. This technique has been further developed and is 2D and 3D clinically applicable now (Daley et al., 2008; McNamara et al., 2010; Yao et al., 2013; Lv et al., 2015).

These fundamental principles of 3D culture and tissue engineering have only recently started to be applied to inner ear research, exemplified lately in the work of Hashino and colleagues who reported generation of mouse and human iPSC organoids (Koehler et al., 2013; Koehler et al., 2017). The design of models that mimic the *in vivo* situation may accelerate the translation of basic discoveries in this particular field, as the organoid systems are a unique tool to study different diseases and regenerative therapies of the inner ear. Moreover, this technique could facilitate exploration of pharmacological interventions.

This review has been undertaken to update the readers about the progress that has been made in understanding the mechanisms behind 3D‐cell fate and is addressed to the field of inner ear regenerative medicine toward the development of inner ear organoids or inner ear structures on a chip. This review highlights the significance of the ECM, the requirements of a 3D ECM, several biomaterials and some advanced production techniques of cell‐laden scaffolds. Although not specifically mentioned, these general paragraphs are relevant to inner ear tissue engineering. Then, the subject is directed toward the human inner ear. Recent studies concerning the generation of inner ear organoids are discussed and we present recommendations for future research, including the prospective use of microfluidic cell culture systems for otic tissue pharmacological research and bioprinting of inner ear structures.

## THE SIGNIFICANCE OF THE ECM

A (stem) cell population *in vivo* is in contact with a 3D ECM that encloses multiple complex components such as different ECM–proteins, for example, glycoproteins and proteoglycans, a variety of endogenous growth factors and multiple neighboring (stem) cell types. In this cell niche all components interact, dependent on the temporal situation of the tissue. This complex system of interactions directly influences important cell functions such as proliferation, differentiation, and migration (Friedl et al., 1998). The building blocks of the ECM are the ECM proteins which determine matrix characteristics such as viscoelasticity, growth factor binding capacity, and permeability to cells and nutrients. Although underestimated for years, the role of the ECM has become more and more prominent during the last two decades (Lewis, 1922; Friedl et al., 1998). To develop cells *in vitro* into organoids or tissues in organoids and/or microfluidic systems, a microenvironment needs to be created that mimics the ECM during embryogenesis of the particular organ or tissue. For that purpose, knowledge about ECM proteins during that period of time is crucial.

Throughout early development, many cells are highly proliferative and move or sprout as organized clusters to new locations with conserved cellular contacts (Friedl and Gilmour, 2009; Rørth, 2011). The cell groups that migrate may nevertheless also migrate while the epithelial organization is maintained. This indicates the existence of partial transitions between epithelial and mesenchymal cell fate (Revenu and Gilmour, 2009). These transitions are coordinated with cell–cell and cell–matrix interaction without losing their tissue structure. Differences in cell adhesion are crucial during cell intercalation, a process whereby cells from different layers lose contact with their neighboring cells and rearrange into a single layer, without losing tissue structure, owing to an increase in surface area. Consequently, differences in information from cell–cell contact but also cell–matrix contact forces groups of cells to differentiate toward various lineages. In addition, live imaging on chick embryos with fluorescent tagged ECM proteins showed a remarkable correspondence between the epiblastic and sub‐epiblastic ECM displacements while morphogenesis proceeded. The simplest explanation is that under certain situations, migratory cells might carry their ECM with them. Although it is uncertain if this observation only takes place during early development, it must be taken into consideration that the ECM at morphogenesis is not necessarily static in one position. These findings are complementary to the work of Evseenko et al., in embroid bodies (EBs), showing the presence and importance of ECM proteins in the cell fate of the cells in the EB. Thus, it has to be taken into consideration that cell fate is not only influenced by mechanical cues caused by cell–cell and cell–matrix contact but also by proteins, that are present in the ECM when an embroid body has formed (Evseenko et al., 2009).

Moreover, during embryogenesis when the different germ layers are formed, the ECM‐composition is diverse. Among them are glycoproteins, proteoglycans, and polysaccharides that exert different biochemical properties (Emerman et al., 2010). By changing various components of the ECM, mimicking different stages in development, a dedicated matrix could be engineered to enable lineage‐specific cell differentiation and organoid development (Emerman et al., 2010).

## SCAFFOLD REQUIREMENTS

### Focal Adhesion

**Figure 1:**
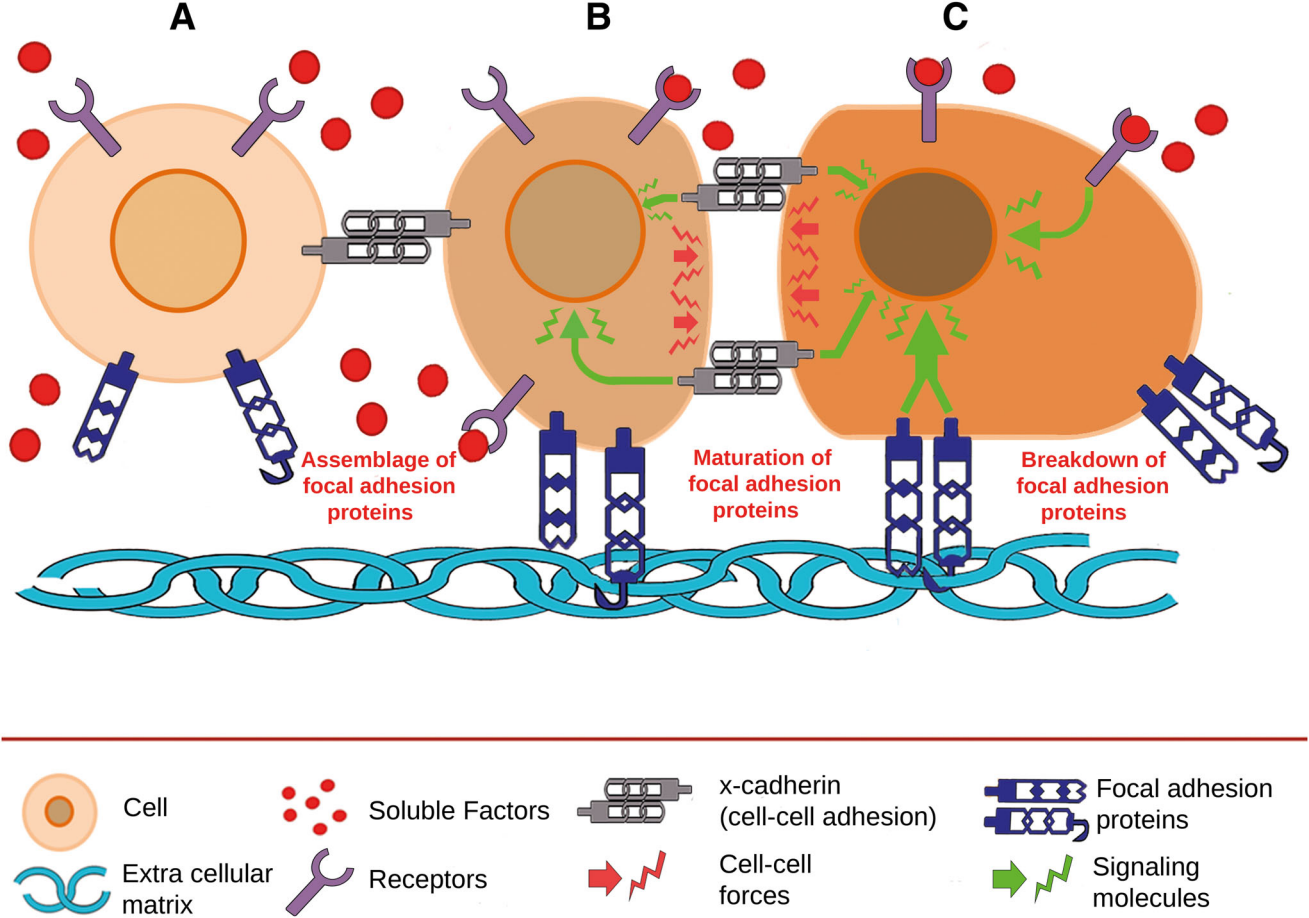
Schematic illustration of the formation and remodeling of focal adhesions.The dynamic process of focal adhesion is influenced by factors such as intercellular adhesion and morphogens. (A) At initial contact of the cells, focal adhesions assemble resulting in binding to specific adhesive motives in the extracellular matrix. (B) Focal and intercellular adhesions are increased, leading to a variety of cellular responses. Pertinent signal transduction pathways are upregulated, in particular, those involved in migratory processes as illustrated by changes in morphology and alignment. These signal transduction pathways may also allow the cell to present specific receptors that bind to corresponding ligands. (C) The concerted effect of all components allows controlled maturation and breakdown of focal adhesions resulting in coincident and coordinated movement of cells along the extracellular matrix. Color differences represent upregulation of processes in the nucleus and cytoplasm.

Focal adhesions are multi‐protein structures that form mechanical links between intracellular actin bundles and the fibers of the extracellular matrix. Focal adhesions are composed of focal adhesion proteins (including integrins, vinculin, talin, F‐actin, and myosin) and signaling molecules (including focal adhesion kinase) and are involved in cell–matrix and cell–cell adhesion interaction (Burridge and Chrzanowska‐Wodnicka, 1996). Focal adhesion complexes are assembled, mature and disassemble to allow mechanosensing leading to a variety of cellular processes (Fig. 1). Cell–matrix adhesion often occurs via cellular integrins, binding to an arginine–glycine–aspartate sequence (the RGD motif) in the matrix (Wang et al., 2013; Kenny and Connelly, 2015). Due to their mechanosensitivity, cells can “feel” the elasticity of the matrix, which subsequently leads to different cellular processes. Engler et al. showed that culturing MSCs on polyacrylamide gels with a bone‐like stiffness directed the cells toward osteogenic differentiation and that culturing the cells on matrices with a brain‐like stiffness led to neuronal differentiation. Moreover, the group demonstrated that differentiation of stem cells occurs via focal adhesion by blocking nonmuscle myosin II with blebbistatin, which is involved in tensioning of actin structures in the cytoskeleton. Therefore, the ability for cells to sense and adapt to different physical microenvironments occurs via focal adhesions and through myosin–actin contractions. Microarray analysis found that myosin contractility plays a critical role in lineage commitment of human MSCs through MAPK pathways and Wnt signaling (Kilian et al., 2010). Cells respond to a stiff matrix with more focal adhesions compared to a soft matrix, leading to better adhesion strength and thus stiffer/tenser cells (Legate et al., 2009). Also, the cell's motility increases on a stiff matrix and cells tend to migrate from soft to stiff matrix (Lo et al., 2000; Zhong and Ji, 2013). The high cell motility at stiff matrices, when compared to matrices of high elasticity, is probably due to the fact that cell velocity slows down when cells have less cell–matrix contacts in low elasticity matrices (Wells, 2008).

*In vivo*, the ECM is also subject to structural changes from cell–matrix interactions. Growing evidence suggests that *in vivo*, ECM strain stiffening is caused by surrounding cells. Strain‐stiffening is defined as an increase in a material's elastic modulus (i.e., the matrix becomes stiffer). In natural polymers, this dynamic process occurs primarily by increased crosslinking of collagen and elastin and is nonlinear (Storm et al., 2005; Lin et al., 2010). This is in contrast to most synthetic gels and rubbers which deform linearly to large strains (e.g., polyacrylamide). This phenomenon of nonlinear elastic response complicates replication in synthetic matrices.

### Mechanotransduction Pathway of ECM to Cell Nucleus

Mechanotransduction via nonmuscle myosin II seems to play a crucial role in ECM sensing, but also other proteins such as those from the Ras superfamily are known to be involved in cytoskeleton formation, cell growth, and transcription regulation (i.e., differentiation; Stenmark and Olkkonen, 2001). A subgroup in the Ras superfamily is the RhoA/ROCK pathway.

RhoA and Rock2 are proteins that are involved in the depolymerization of actin fibers and contribute to changes in shape, adhesion, and polarity of the cell. By blocking the RhoA/ROCK and Focal adhesion kinase (FAK) proteins with specific inhibitors, changes in cytoskeletal organization in human MSCs were observed. Studies have also shown that RhoA/ROCK and FAK are involved in mechanical stretching of the cell (Hirata et al., 2008; Maharam et al., 2015).

In addition, mechanotransduction could also be initiated through cell–cell adhesion by cadherins instead of classical ECM–cell signaling. Cadherins are transmembrane proteins that are connected to focal adhesion proteins through an alpha‐catenin/beta‐catenin complex which directly binds to the actin cytoskeleton (Fig. 1; Leckband and De Rooij, 2014).

During mechanotransduction, β‐catenins are also involved in Wnt‐signaling. When epithelial‐cadherins (E‐cadherins) are sequestered due to a strong cell–cell adhesion, less β‐catenins are available for nuclear localization and thus less Wnt target genes can be transcribed (Jamora and Fuchs, 2002). Wnt/beta‐catenin pathway is a mechanism that is involved in proliferation, differentiation, and many other cell functions. Wnt/β‐catenin responses can also be affected by mechanotransduction *via* cellular membrane integrins. Through the integrin/FAK pathway, beta‐catenin accumulates and shuttles to the nucleus, which can promote Wnt1 gene expression (Du et al., 2011).

Alternatively, it has been shown that Rho kinase 2 (ROCK2) can also be responsible for the cross talk between β‐catenin, integrins, and cadherins, activating Wnt target genes (Samuel et al., 2011). How ROCK2 sequesters β‐catenin to the nucleus without effecting E‐cadherins still remains unclear.

### Topography of the ECM

As mentioned above, cells actively sense their environment through ECM–cell and cell–cell contact. However, another element appears also to be of importance: matrix topography. When cells reside in their natural niche, they encounter an ECM of which the fiber‐architecture varies in nano‐ and microscale. Differences in ECM architecture on the cellular level alter cell–matrix contact, which is another way to control cell–ECM interaction. One of the techniques used is creating micropatterned islands. These adhesive islands are patterned and can be created by using microcontact printing and are substrates of a defined shape and size on a background that otherwise prevents cell adhesion. The shape of these micropatterned islands can be manipulated to control cell spreading and can also be used to mimic the physiological spatial confinement thereby creating new shapes of substrates with a constant surface adhesion area. These surfaces can be coated with ECM in a 2D fashion where single human stem cells can reside. Stem cells favor differentiation when placed onto circular/spherical substrates. This indicates that the amount and the composition of ECM is not the only initiator for differentiation of stem cells but that ECM/cell shape also takes a crucial role in stem cell fate.

In human MSCs, the intracellular response to mechanotransduction is different when seeded on micropatterned islands when compared to a nonpatterned substrate. It has also been shown that the degree of cell‐spreading on these islands by human MSCs determined their differential expression (McBeath et al., 2004). It has been described how a smaller surface area forces human MSC to spread to a lesser extent, resulting in a globular cell morphology, which promoted adipogenesis (McBeath et al., 2004). This in accordance with the report of Kilian et al. (2010) who demonstrated that the geometric features of MSCs can coordinate mechanochemical signals and paracrine/autocrine factors, directing the cells toward a specific fate (Kilian et al., 2010). Also, changing the topography of the matrix, for example, the depth of the cell substrate influences the rate and lineage of differentiation (Zouani et al., 2012).

Usage of a ridge groove pattern on poly(dimethylsiloxane) (PDMS) substrate has an effect on neuron development and further differentiation into downstream lineages (Mahoney et al., 2005). PC12 cells, a neuroblastic cell line, *in vitro* placed in microgrooves of polyimide causes improved neurite outgrowth and while for glia (Schwann cells), the groove width was of importance for cell alignment (Miller et al., 2001; Lietz et al., 2006). Human MSCs were aligned and elongated along the micro‐grooved poly(methyl methacrylate) (PMMA) scaffold, immunostaining and RT‐PCR showed upregulated neural expression of MAP2, GFAP, and TUJ1 (Yim, 2007). In addition, scaffold‐based delivery of neuronal induction factors to human MSCs can result in significant changes in neuronal morphology and expression of the cells when compared to controls (Yiang et al., 2012). Human MSCs on a micro‐grooved pattern of 1 μm PDMS showed an increase of intracellular calcium which is needed for neurite outgrowth whereas a 4 μm groove had no calcium response (Rusanescu et al., 1995; Kim et al., 2008). Beduer et al. (2012) found that neural stem cells, seeded on thin micro‐grooved channels, stopped proliferating and started differentiation. This resulted in more polarized neurons whereas large microgrooves lead to a higher amount of neurite outgrowth. More neurite outgrowth is essential for immature neurons for further differentiation into neuronal lineages (Beduer et al., 2012). It seems that a neuronal progenitor favors to grow near the ridge of a groove in order to stimulate neurite development (Beduer et al., 2012). Interestingly, when naïve human ESCs are seeded on a 350 nm ridge grove polyurethane acrylate (PUA) substrate, the cells differentiated into a neuronal lineage in 5 days without the addition of neuronal growth factors. These human ESCs were able to differentiate into neurons but not into a glial lineage (Lee et al., 2010). However, this study did not mention the yield of neurons, making the outcome of the proposed method rather uncertain. Other studies involving 400 nm electrospun nanofibers resulted in a high number of neuroprogenitor cells and yielded many neuronal cells and mature motor neurons from human ESC (Shahbazi et al., 2011). Chan et al. found in stabilized culture conditions that 2 μm grooves resulted in the best neuronal differentiation for both human ESCs and human iPSCs. In addition to surface topographical features, which can direct cells toward neuronal differentiation, cell geometry, and cell–cell connections are also important issues. When cell density increases, neurite outgrowth, and alignment is very different for the various grooves geometries (Beduer et al., 2012). In this way, neuronal networks with a controlled architecture can be designed. Altogether it has clearly been shown that the use of topographical cues via ridge/groove patterns or fibers can drive stem cells into a neuronal lineage but the exact mechanism still remains unknown.

As demonstrated by Engler et al. MSCs seeded on matrices with a soft substrate, in which the elasticity ranges from 0.1 to 1 kPa, favor neuronal differentiation. Further studies in stem cell fate of NSCs on a synthetic, interfacial hydrogel culture system, showed that the best neuronal differentiation takes place at scaffold stiffness of 0.5 kPa which is similar to the stiffness of brain tissue (Saha et al., 2008). Furthermore, stiffness of 0.1–0.5 kPa leads to differentiation to neurons while a stiffer matrix of 1–10 kPa promotes glial differentiation. Also, NSCs on a 3D alginate hydrogel enhanced TUB3 expression on the lowest stiffness (0.5 kPa; Banerjee et al., 2009). Interestingly, there are stiffness differences within the hippocampus which may indicate that further differentiation into the neuronal lineage is dependent on small differences in ECM stiffness (Keung et al., 2011). Studies with a 3D scaffold of hyaluronic acid and PuraMatrix™ showed that when methacrylation of hyaluronic acid was decreased, neurite outgrowth from dorsal root ganglia (DRG) explants was more prominent. Methacrylation of hyaluronic acid causes degradation of crosslinking which corresponds with a certain degree of permeability (Khoshakhlagh and Moore, 2015). To ensure permeability in low elastic matrices, bio‐active hydrogels with microchannels were produced without changing the rigidity of the gel (Lee et al., 2015). In these gels, MSC adhere to the gel and differentiate into neurons and glial cells exclusively in the microchannels.

On soft matrices, neurogenic differentiation can be modulated by inhibition of the BMP/SMAD pathway (Du et al., 2011). Bone marrow stem cells seeded on soft matrices express less surface integrins than on stiff matrices. This is probably due to unstable integrin–ligand binding on soft substrates. This unstable integrin–ECM binding results in internalization of integrins which also affects the localization of bone morphogenetic protein (BMP) receptors. As a result, BMP receptor endocytosis occurs which in turn causes repression of BMP/SMAD pathway. The inhibition of SMAD subsequently leads to upregulation of neuronal genes including MAP2, neurofilament, and nestin.

For many years, the creation of matrices focused on sufficient permeability through the whole scaffold. However, increasing evidence supports the contribution of substrate stiffness and topography on stem cell differentiation. It is therefore clear that to design the optimal matrix for targeted stem cell differentiation, a balance needs to be found between substrate rigidity, permeability, and topography (Lee et al., 2015). It is recommended to use an algorithm‐based topographical biomaterial library to find the ideal substrate topography for instructing cell fate (Unadkat et al., 2011; Bennett et al., 2016).

## BIOMATERIALS

To study cell culture *in vitro*, several alternatives are presented in the literature that describes how to derive an ECM environment mimicking the *in vivo* situation (Murphy et al., 2017). The complexity of native ECM is said to be represented best by Matrigel™ (Gelain et al., 2006), although often hydrogels consisting of a single ECM component, such as collagen, are applied (Kleinman and Martin, 2005). Ideally, a pertinent scaffold should be built which provides structural support to cells during their lifetime and which can influence (stem) cell fate as well. This means that, depending on the application, some of the natural polymers have to be treated chemically in order to improve/change their stability/mechanical properties. Moreover, not all of these polymers contain adhesive sequences that may take over the role of the *in vivo* ECM for *in vitro* cell development purposes. Therefore, current practice is to blend two or more polymers and to allow covalent binding of adhesive molecules. For each particular tissue engineering purpose, it is possible to produce tailor‐made matrices. In the process of designing a scaffold, several aspects should be addressed. Important features are biocompatibility and safety to both natural and synthetic polymers. Hydrogels to be used as a matrix should not evoke an inflammatory response and may not contain harmful substances such as pathogens. Moreover, in particular applications for instance bioprinting, degradability, and injectability can be an issue.

### Natural Polymers

#### *Proteins: Fibrin, silk fibroin, fibronectin, laminin, and collagen*

Fibrinogen is an injectable precursor for fibrin and can be generated from human plasma, providing autologous ECM usable for organoid‐ or organ‐on‐chip research. Polymerization of this protein is initiated upon thrombin addition resulting in a very flexible though mechanically weak fibrin meshwork (Kneser et al., 2005). It is advantageous that a fibrin network is highly elastic and very stable and stiffening is easily achieved by applying tension or compression, which makes fibrin very suitable for bioprinting (Benning et al., 2017).

Silk fibroin has proven a useful protein for developing neuronal systems. This protein derived from cocoons of the silkworm among others, forms packed β‐sheets that do not need crosslinking for gelation (Nagarkar et al., 2010). In addition to this physical crosslink through β‐sheet formation, silk fibroin can also be chemically crosslinked (Elliott et al., 2015). In such a way, hydrogel formation and mechanical properties can be controlled. Silk fibroin stiffness is comparable to brain tissue and the stability outlasts the structural integrity of fibrin and collagen. To acquire adhesive properties additional molecules have to be added (Hopkins et al., 2013). Due to the soft silk fibroin, this matrix is very suitable for bioprinting. This has recently been shown by Das et al. (2015) who used silk–gelatin based bio‐ink loaded with mesenchymal stem cells and proved multilineage differentiation and high cell viability in the matrices.

Fibronectin is a glycoprotein which is involved in cell migration in the developing central nervous system (Stettler and Galileo, 2004). Fibronectin also has a significant role in tissue repair because of its cell adhesion properties and its ability to sequester nutrients and growth factors (Venstrom and Reichardt, 1993). Moreover, fibronectin can be secreted by different types of cells, which increases the therapeutic potential of this glycoprotein (Mao and Schwarzbauer, 2005). This has recently been shown by Roberts et al. (2017) who utilized a solvent degradable hollow fiber membrane as a cell culture platform for 3T3 fibroblasts. In such a model, the patient's own cells could be used to produce an appropriate matrix.

Laminin is a heterotrimeric glycoprotein composed of alpha, beta, and gamma subunits. Laminin organizes in sheet‐like polymers which facilitate both cell proliferation and movement during development. In the mature central nervous system, for unknown reasons, laminin is mainly present in regenerative niches and along blood vessels and remains moderately high (Liesi, 1985). During development and wound healing, laminin promotes neuronal attachment and biosynthesis. Neuronal attachment is procured by the amino acid sequence isoleucine, lysine, valine, alanine, and valine (IKVAV; see also “Adhesive (peptide) sequences” section). It has been reported that α1β1γ1‐laminin hydrogel can be produced as an injectable hydrogel without the need of an initiator for gelation (Francisco et al., 2013).

Collagen fibrils consist of predominantly glycine repeats buffered by two amino acid moieties, often proline and hydroxyproline residues, and provide structure whereas both collagen and laminin possess cell adhesive properties. To create a 3D environment, the cells can be mixed with a liquid precursor after which the suspension is allowed to polymerize, encapsulating the cells. The disadvantage is that the collagen precursor is liquid below 4°C, a condition harmful for cells. Nevertheless, systems using collagen as a matrix in a microfluidic system can be of great use to study cellular migratory behavior, for example, invasion of tumor cells *via* blood vessels or neurite outgrowth toward growth factor gradients (Kothapalli et al., 2011; Chonan et al., 2017)

#### *Matrigel*

Matrigel™ is most often reported in research of neuronal 3D cell culture. This hydrogel is derived from basement cell membranes from Engelbreth–Holm–Swarm mouse sarcoma. Matrigel™ contains all major components such as collagen and laminin of the *in vivo* ECM including growth factors and cytokines (Kleinman and Martin, 2005). A great advantage is that it is an approved commercial product. It has been shown that during cultivation of inner ear hair cells when comparing collagen type I “single component” gel to Matrigel™, the latter outperformed in hair cell density and viability (Spencer et al., 2008). Clearly, the fact that Matrigel™ is rich in ECM proteins made this scaffold perform better than just collagen type I (Spencer et al., 2008). The study also makes a comparison with a synthetic scaffold (PuraMatrix™, see self‐assembling peptides), the latter turns out to be the ultimate matrix of choice. In spite of its advantages, Matrigel™ should be used with care, because the gel harbors an undefined number of growth factors that may be advantageous, but may also vary a lot between different batches which could lead to inconsistent or irreproducible results (Kleinman and Martin, 2005; Hughes et al., 2010). The same holds true for single component collagen gel, of which biochemical and mechanical properties are subject to batch‐to‐batch variations. In addition, Matrigel™ is mouse‐derived and is neither safe nor practical for human studies. Collagen and Matrigel™ hydrogels are commonly used in injection studies although it has to be taken into account that their sol–gel transitions require dramatic changes in solution parameters (i.e., pH for collagen and temperature for both collagen and Matrigel™). When cells have to be encapsulated, these nonphysiological conditions may be harmful (Parisi‐Amon et al., 2013).

#### *Polysaccharides: hyaluronic acid, alginate, and chitosan*

Another scaffold derived from nature and frequently used in central nervous system research is hyaluronic acid (HA) which is derived from chicken combs or vitreous humor (Hou et al., 2005; Rosa et al., 2012). HA is a glycosaminoglycan (GAG) and a major component of the natural ECM. Because HA has the same mechanical properties (i.e., stiffness) as brain tissue, this matrix facilitates, when injected into the brain, stem cell engraftment and survival. Moreover, it stimulates angiogenesis without creating scar formation or tissue rejection (Hou et al., 2005; Potter et al., 2008) and is for these qualities already widely used in the field of skin rejuvenation (Bass, 2015). A disadvantage is that, in physiological environments, HA is subjected to various degradation processes due to hydrolysis and enzymatic hydrolysis by naturally occurring hyaluronidase. Different strategies such as crosslinking or conjugation have been used to stabilize HA and maintaining at the same time its fundamental properties (Borzacchiello et al., 2015). Another disadvantage of HA is that GAGs do not function as adhesion molecules but solely add compression strength, lubrication, and hydration to the ECM (Alberts et al., 2001). HA is therefore often combined with adhesion molecules such as poly‐d‐lysine (PDL), RGD‐containing collagen or heparin (Hou et al., 2005; Potter et al., 2008).

Like HA, alginate, and chitosan are polysaccharides and do not contain adhesive motifs. For that reason, these polysaccharides are often blended with adhesive polymers (Hou et al., 2005; Gwak et al., 2007; Zhong et al., 2010). Alginate consists of mannuronic and glucuronic acid units conferring a variable 3D structure, whereas chitosan is built up solely from β‐(1–4)‐d‐glucosamine monomers. The advantage of alginate is that it is injectable, which makes it suitable for bioprinting (Axpe and Oyen, 2016). However, the sol–gel transition of the anionic alginate is achieved by changing the ionic strength of the solution, which challenges cells with nonphysiological conditions during gelation (Ionita et al., 2015). The applications of natural and biodegradable chitosan have been limited by the poor solubility of native chitosan in neutral pH solution. This has been addressed by Li et al. (2015) who incorporated a low‐dose UV crosslinking ability in chitosan, allowing fabrication of hydrogels in which cells remain viable and can be incorporated at physiological pH conditions (Li et al., 2015). In Table 1, all natural polymers, their advantages and disadvantages concerning 3D culturing are summarized.

**Table 1:** An overview of “Biomaterials” section, natural polymers.

Natural* ^,^ **		Pro	Contra
(Glyco)proteins	Fibrin	‐ Injectable ‐ Suitable for bioprinting ‐ High elasticity ‐ High stability ‐ Cell adhesive sequence***	Mechanically weak
	Silk fibroin	‐ Outlasts structural integrity of Fibrin and collagen ‐ Controllable mechanical properties ‐ Suitable for bioprinting, mixture with gelatin ‐ Stiffness comparable to brain tissue	Adhesive properties have to be added
	Fibronectin	‐ Cell adhesion properties *** ‐ Ability to sequester nutrients and growth factors ‐ Produced by patients own cells ‐ Used as a coating of synthetic polymers	
	Laminin	‐ Injectable ‐ No need for gelation initiator ‐ Cell adhesive sequence both RGD and IKVAV***	
	Collagen	‐ Injectable ‐ Cell adhesive sequence*** ‐ Successful in microfluidic systems	Harmful cell encapsulation conditions
Matrigel™		‐ Injectable ‐ Approved commercial product ‐ Several ECM proteins and growth factors present ‐ cell adhesion properties	Harmful cell encapsulation conditions
Polysaccharides	Hyaluronic acid	‐ Adds compression strength, lubrication, and hydration to ECM ‐ injectable ‐ Control of permeability by methacrylation ‐ Stiffness comparable to brain tissue ‐ Stimulates angiogenesis	No cell adhesion motifs
	Alginate	‐ Injectable ‐ Suitable for bioprinting	‐ No cell adhesion motifs ‐ Variable 3D structure ‐ Nonphysiological conditions during gelation
	Chitosan	‐ Antimicrobial anti‐inflammatory ‐ Physiological cell encapsulation conditions when modified	‐ No cell adhesion motifs ‐ Poor solubility

Background information is indicated by symbols.

* In almost all natural polymers there is a potential risk of pathogen transmission, because most of them are derived from animal sources.

** Most natural polymers are subject to batch‐to‐batch variation, inhomogeneity and they are easily degraded in a physiological environment.

*** RGD adhesive motif may not always be exposed for integrin binding, this depends on the protein conformation (Bellis, 2011).

### Synthetic Polymers

Compared to natural polymers, synthetic polymers have the advantage that the purity of the building blocks is defined which implies that the product can be standardized and that a stable composition of the hydrogel in every batch can be guaranteed. The fact that multiple constituents of the ECM should be present for a true mimic of the *in vivo* situation, holds as much for the synthetic as for the natural polymers. In the case of synthetic polymers, these requirements often can be introduced in a controlled fashion if a specific synthetic route is followed. On top of that, an ideal 3D culture matrix consists of a biomaterial with fibers and pore sizes smaller than the diameter of an average cell to ensure optimal cell–cell contact and to ensure delivery of nutrients. Especially self‐assembling peptides demonstrate the capability for tailor‐made functionality and quality by design. Interesting developments in the fabrication of scaffolds show potential to produce these dimensions on demand (see: electrospinning).

#### *Polyethers, polyesters, polyacrylates, and polyacrylamide*

Many of the synthetic scaffolds are made of single fibers such as poly (2‐hydroxyethyl methacrylate) (PHEMA), poly(ethylene glycol) (PEG), poly(acrylamide) (PA), and poly(epsilon‐caprolactone) (PCL) (Lee and Mooney, 2001; Rampichova et al., 2012). These microfibers often have a diameter of about 10–50 μm which is similar to the diameter of most cells cultured *in vitro*. Thus, when culturing cells with these microfiber scaffolds, different cells can adhere to one large fiber at the same time, so the cells are still in a 2D environment. Moreover, the pore size of this kind of synthetic scaffold is often 1,000 to 10,000 times larger than the microfiber itself. A consequence of engineering such a scaffold with relatively large pore size is that extracellular factors such as hormones and growth factors tend to diffuse away, reducing one of the physiological properties of the ECM. On the contrary, when a hydrogel is heavily crosslinked, the permeability of the gel may be compromised (Lee et al., 2015).

Although polyethers, esters, and acrylates have been proven to be helpful in cell culture studies creating a hydrated environment of certain elasticity, their adhesive capacity generally has to be enhanced by the (covalent) attachment of adhesive elements or coating with *e.g*., a fibronectin layer.

Special attention was drawn to poly(acrylamide) (PA) gels of different elasticity when Engler described them in his seminal paper (2006) in which, depending on the stiffness of PA gels, MSCs differentiated toward osteogenic or neuronal cell types (Caliari et al., 2016). PA hydrogels are produced by reacting acrylamide monomer and a crosslinker, bisacrylamide, usually in the presence of ammonium persulfate and tetramethylethylenediamine. Protein conjugation to the PA hydrogel surface to enable cell attachment is applicable and well‐established protocols for the fabrication of PA hydrogels with tunable stiffness and coupling of proteins are available (Tse and Engler, 2010; Trappmann et al., 2012). Therefore, PA is ideally suited for mechanobiology studies in which hydrogel stiffness needs to be finely controlled. A disadvantage is that PA hydrogels do not inherently interact with cell surface receptors or integrins. One major disadvantage is that PA cannot be used to encapsulate cells in 3D, because of the toxicity of the hydrogel precursors (Caliari et al., 2016).

#### *Self‐assembling peptides*

Polypeptides constitute a different class of synthetic polymers. An effective way to generate nanofiber scaffolds is the utilization of self‐assembling amphiphilic peptides. Amphiphilic peptides are arranged as periodic repeats of hydrophilic and hydrophobic amino acids. When submerged in water under the right circumstances, such as a low pH or an increasing salt concentration, the hydrophobic part will coil up, exposing the hydrophilic part, followed by assembly with other (coiled) peptides into an intertwined structure. Hence, no complex technique such as electrospinning is needed to create a nanofiber scaffold. Most self‐assembling peptides designed for tissue engineering, form bilayers in β‐sheet fiber structures in water (Kim et al., 2015). Nanofibers of self‐assembling peptides typically have a diameter of approximately 10 nm, a 1,000 times smaller than synthetic microfibers (Holmes et al., 2000). These nanofiber peptides are designed at an amino acid level so they can easily be modified, allowing for covalent functionalization with alternative adhesive motifs. The concentration and position of these motifs in the final scaffold can be meticulously controlled and finally, (stem) cells can be encapsulated in injectable scaffolds of which every constituent is determined. An architecture like this is hard to achieve by means of other synthetic techniques, while therapeutic perspectives of these pure gels are favored over natural matrices that are undefined and may contain harmful substances/pathogens (Gelain et al., 2006; Koutsopoulos, 2016).

RADA16 (PuraMatrix™) is a widely studied and commercially available self‐assembling amphiphilic peptide that is designed to contain periodic repeats of positively charged arginine (R) the hydrophobic alanine (A) and negative aspartic acid (D). RADA16 self‐assembly is initiated by changes in ionic strength. Gelation occurs at physiological pH dependent on the salt concentration in the culture medium. Temperature changes, usually needed for gelation of collagen and Matrigel™, are not required. The resulting nanofibers are ordered in a similar way to native ECM, which makes PuraMatrix™ an attractive hydrogel for neuronal differentiation studies. Moreover, PuraMatrix™ incorporates an adhesion motif and has been implicated to support neuronal adhesion, promote neurite outgrowth, and contribute synaptic formation between the neurons in P12 cells (Holmes et al., 2000). Formation of a vascular network, important for transport of nutrients and oxygen in sustaining organoids, was investigated comparing the vasculature formed by endothelial stem cells and MSCs in PuraMatrix™, fibrin, and collagen type I (Allen et al., 2011). The vascular density turned out to be far greater in PuraMatrix™, a result attributed to this gel because it is more compliable (soft) than fibrin and collagen gels. In Table 2, all synthetic polymers, their advantages, and disadvantages concerning 3D culturing are summarized.

**Table 2:** An overview of “Biomaterials” section, synthetic polymers.

Synthetic# ^,^ ##		Pro	Contra
			
Polyethers	Polyethyleneglycol (PEG)	‐ FDA approved ‐ High water content hydrogel after crosslinking	‐ Adhesive capacity has to be introduced ‐ Biodegradability has to be improved###
Polyesters	Poly(epsiloncaprolacton) (PCL)	‐ FDA approved ‐ Biodegradable (lipases esterases)	‐ Adhesive capacity has to be introduced ‐ Relatively hydrophobic
Polyacrylates	Poly(2‐hydroxyethylmethacrylate) (PHEMA)	‐Functional groups for introducing adhesive functionality ‐ Well hydrated ‐ Biodegradable (75% in 17 days)	Adhesive capacity has to be introduced
Polyacrylamides	Poly(acrylamide) (PA)	‐ Tunable stiffness ‐ Protocols for controlled protein coupling available	‐ Adhesive capacity has to be introduced ‐ Hydrogel precursor is toxic (= not biodegradable)
Polypeptides	Puramatrix™	‐ Intrinsic nanofiber scaffold hence 3D ‐ Encapsulation of cells under physiological conditions ‐ Easily modified at amino acid level with adhesive sequence ‐ Injectable ‐ Biodegradable ‐ Softer than fibrin and collagen gels	

Background information is indicated by symbols.

# Synthetic polymers are more strictly defined than their natural counterparts and purity of components may guarantee that no pathogens are present.

## Often microfibers are frequently used, but actually, this is a 2D environment.

### For instance, by incorporating matrix metalloproteinase cleavable motifs.

### Adhesive (Peptide) Sequences

The presence of RGD or RGD‐like adhesive units in silk fibroin, fibronectin, and PuraMatrix™ was already discussed. Techniques using phages have revealed other short peptide epitopes, which also function as adhesive units and among them are those relevant to neural tissue engineering (Koss and Unsworth, 2016). One of these epitopes is the isoleucine–lysine–valine–alanine–valine (IKVAV) peptide, derived from the α‐1 chain of laminin. It has been shown that hydrogels incorporated with IKVAV hydrogel promote adhesion of PC12 cells, direct neurite sprouting and neurite cell growth (Kam et al., 2002; Silva et al., 2004; Wu et al., 2006). Silva et al. (2004) found that neural progenitor cells seeded in IKVAV hydrogel very rapidly differentiated into neurons while no astrocyte development was found. The effect of IKVAV in neuronal differentiation and tissue engineering was assessed by comparing natural silk fibroin hydrogel covalently modified with IKVAV with unmodified hydrogel. The study showed that the hydrogel modified with IKVAV can increase cell viability and neuronal differentiation when compared with cells encapsulated with silk fibroin hydrogel alone (Sun et al., 2017). Recently, studies with synthetic PuraMatrix™ covalently combined with the IKVAV motif showed promising results by differentiating NSCs toward neurons and astrocytes without the addition of growth factors (Sun et al., 2016).

The flexibility of peptide synthesis allows scaffold design at the amino acid level, Sahab Negah et al. (2017), for example, incorporated a glycine and serine residue between the RADA16 and IKVAV peptide sequences to grant the peptide strength.

### Mixing/Blending

Both natural and synthetic polymers have their advantages and limitations. While a natural polymer such as collagen or HA possesses several properties like the native ECM, in their isolated form they lack suitable properties for cell survival, proliferation, or differentiation, and come with bio‐variation which impedes reproducing a consistent artificial ECM. On the other hand, a synthetic polymer has defined mechanical properties and composition. Both natural and synthetic polymers need to be enriched with adhesive peptides, normally present in a native matrix. The polysaccharide alginate, for example, has been mixed with collagen which contains adhesive motifs (Lawson et al., 2004). Coassembly of an RGD with an IKVAV epitope combines two adhesive sequences in an attempt to arrive at a more complex ECM mimic (Horgan et al., 2016). Controlled nanofibril formation was demonstrated resulting in a self‐assembling peptide (SAP) with bioactive characteristics and rheological properties that lie in between gel properties of the separate peptides alone. Thus, apart from improving adhesive properties, mixing one polymer with another adjusts the mechanical properties of the resulting gel. Especially in the case of synthetic polymers, a higher degree of homogeneity is to be expected.

### Requirements of Morphogens

Cell fate can directly be influenced by the cell's physical microenvironment, but can also be dictated by para/autocrine signaling through cellular cues such as growth factors, trophic factors, and cyto‐ or chemokines. Stem cells have to decide between proliferation or self‐renewal and differentiation into a more specified type of cell. Extracellular inducers involved in cellular differentiation are mostly well‐defined molecules. Most inducers are very potent, but still very little is known about the temporal dynamics and expression level of the individual compounds when used *in vitro* to ensure differentiation. Research is ongoing in guiding stem cells toward specification while using phased procedures. For instance, Wichterle et al. (2002) described a detailed protocol to guide unspecialized ectodermal cells to motor neurons in a multi‐step manner. In this protocol, induction was started using BMP/Wnt/FGF signaling where after RA was added. SHH was used to terminally differentiate neuronal cells into motor neurons.

## ADVANCED SCAFFOLD PRODUCTION TECHNIQUES

### Electrospinning of Nanofibers

A relatively modern technique called electrospinning allows the generation of fibers at a nanoscale and is often used to produce scaffolds for tissue engineering. Electrospinning is a process in which ultrathin, multifilament fibers with diameters in the nanometer range are created by spinning and manipulating streams of electrically charged polymers in a strong magnetic or electric field, used to make specialized fabrics, such as those in space suits (Cadogan and Shook, 2011). Many polymers can be used to electrospin nanofibers and this technique is not limited to synthetic polymers. Naturally derived polymers such as collagen, gelatin, and hyaluronic acid can also be electrospun and promising results have been achieved by using wheat protein nanofibers (Woerdeman et al., 2005). The advantage of using electrospun scaffolds is that the major chemical, physical (pore size), and mechanical (elasticity) properties of the ECM can be modified (Lim and Mao, 2009). ESCs cultured in a PCL‐electrospun nanofiber 3D scaffold, often aggregate into EBs and after addition of the differentiation factor, retinoic acid (RA), these EBs can give rise to a variety of different neural cell types: neurons, astrocytes, and oligodendrocytes (Xie et al., 2009). Moreover, when culturing rat NSCs on an electrospun nanofiber PCL scaffold, the stem cells differentiated into oligodendrocytes without the addition of neuronal growth factors (Nisbet et al., 2008). This indicates that a nanofiber scaffold that contains PCL polymers is capable to initiate differentiation of NSCs directly, which emphasizes the importance of choosing the right material to employ the pertinent scaffold in 3D cell culture (Zhou et al., 2010).

### Bioprinting

**Figure 2:**
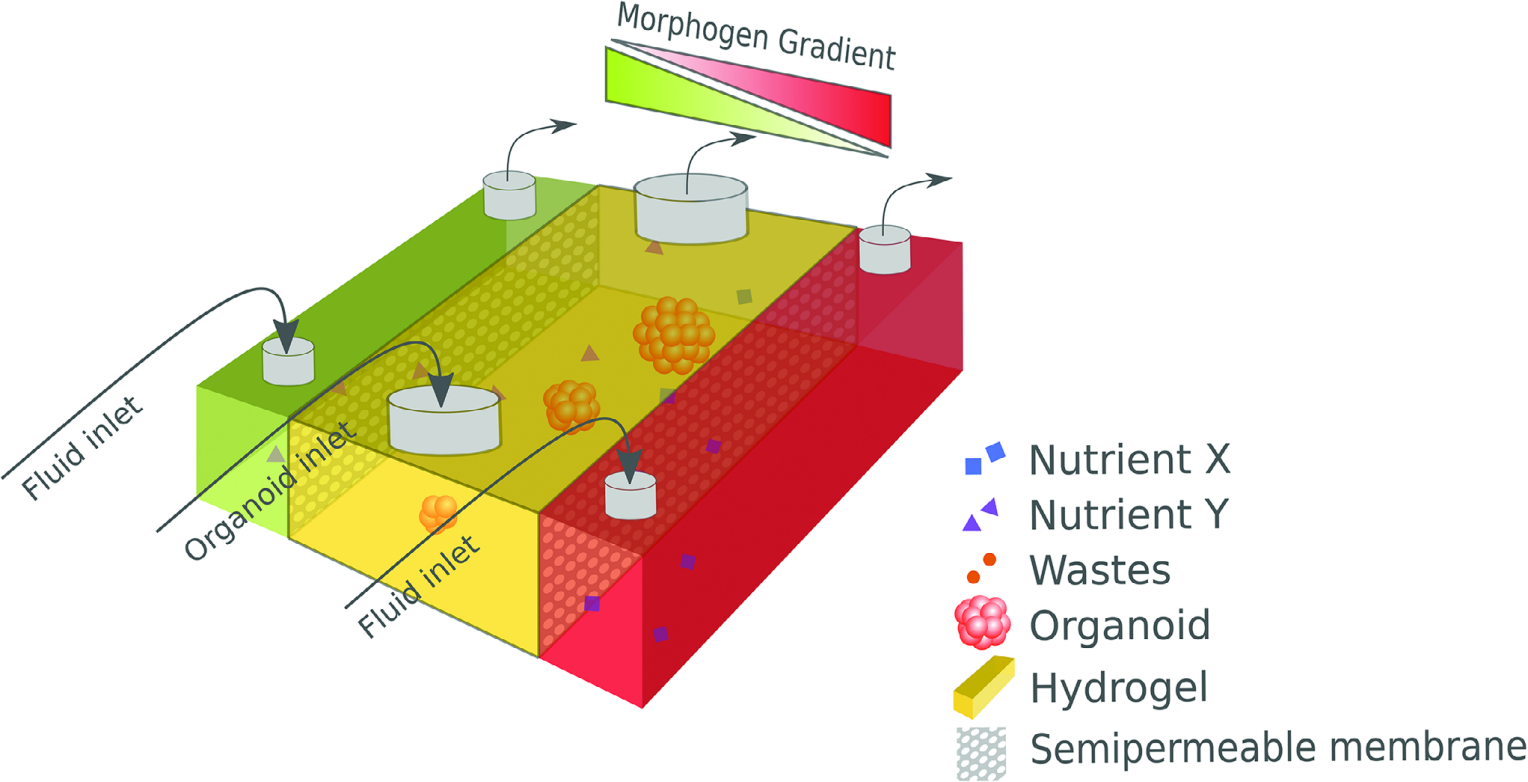
Schematic representation of an organoid or organ‐on‐chip model. Inlet: In a controlled way, special nutrients and morphogens can flow through the chambers. Diffusion of molecules from the two lateral chambers to the central chamber, leading to binding of these molecules to the extracellular matrix and membrane receptors, is facilitated by the semipermeable membrane between the two lateral chambers and the central chamber. Using this model, gradients of different ligands and/or morphogens can be established in the central chamber. The central chamber can be filled with (injectable) extracellular matrix containing organoids or mini‐organs. Outlet: Medium containing CO2 and metabolic waste products can be removed from the culture via the outlets.

3D bioprinting is a method that involves the automated, spatially controlled deposition of cells and/or cell‐containing materials (often referred to as “bio‐ink”) in defined patterns in 3D space (Nguyen and Pentoney Jr., 2017). Computer‐aided design technology is combined with various techniques such as droplet‐, extrusion‐, or laser‐based bioprinting to create tissue constructs. Various materials and techniques are combined to maximize the benefits. The results are promising, successful bioprinting of cartilage, bone, cardiac, nervous, liver, and vascular tissue has been reported (Leberfinger et al., 2017). However, a major limitation to clinical translation is building large‐scale vascularized constructs. Efforts have been made in the field of bioprinting by vascularizing tissue constructs by using microfluidics. Integrating 3D printed tissue to a microfluidic system makes it possible to create a multi organ‐on‐chip (Fig. 2). Lee and Cho (2016) successfully bioprinted a liver‐on‐a‐chip by combining a microfluidic chip with a bioprinted hepatocyte collagen hydrogel (Lee and Cho, 2016). The bioprinted method showed an increased spatial heterogeneity compared to a multistep chip fabrication such as secondary cell seeding. Generally, when bioprinting is combined with microfluidic devices, the bioprinted tissue structure is more likely to mimic the native tissue and provides a better response in organ‐on‐chip applications (Jang, 2017).

However, many challenges must be overcome before bioprinting technology is used routinely in a clinical setting (Leberfinger et al., 2017).

## FOCUSSING ON THE INNER EAR

To generate inner ear tissue, whether in organoids or in a microfluidic system, early developmental processes are of importance from the onset of organoid culture. The early period of inner ear development has been excellently reviewed and hence, within the scope of organoid generation, we will briefly focus on the early regionalization of the ectoderm and the later specification of placodal‐derived cell types (Ohyama et al., 2007; Patthey et al., 2014; Moody and LaMantia, 2015; Basch et al., 2016).

### Early Development of the Inner Ear Cell Types

Most research about early phases of inner ear development has been performed in chicks and mice; but it seems that placode formation is evolutionary conserved because those results were mostly comparable, which led to the presumption that in the human inner ear placode formation and development move along similar pathways (Uchikawa et al., 2003). Later developmental processes are different which is clearly shown by the difference in the start of hearing between mice and humans; while young mice hear after birth, the human fetus is already capable of hearing around the third trimester of pregnancy (Locher, 2005; Freyer et al., 2011; Fuchs and Tucker, 2015).

It has been shown that inner ear cells come from three different sources: the otic placode, neuroepithelial cells, and the neural crest, respectively (Freyer et al., 2011). Placode cells and neural crest remain in close proximity throughout their development and interact repeatedly in a reciprocal manner (Steventon et al., 2014). Placode cells are responsible for the sensory epithelium and the spiral ganglion neurons. The descendants of neuroepithelial cells account for a substantial part of the cell population of the otic vesicle and are specifically present in proneurosensory domains. Neural crest cells develop into the glia cells and melanocytes, which are present in the stria vascularis. Upon their early contact during otic placode formation, neural crest and placode progenitors form transient contacts that involve the accumulation of N‐cadherin. These contacts result in a local disruption of focal adhesions of placodal cells to the extracellular matrix. It has been suggested that neural crest cells and placode cells being successively attracting and rejecting each other build structures which eventually form the inner ear during development (Steventon et al., 2014).

### Inner Ear Development and the ECM

There are a few reports in the literature of different ECM molecules during these early stages of mammalian inner ear formation but to the best of our knowledge, no report is available of the viscoelasticity of the matrix during this period of time (Hilfer and Randolph, 1993; Visconti and Hilfer, 2002). In general, during early otic placode formation in vertebrates, the otic placode becomes attached to the neural ectoderm through a single layer of laminin (Hilfer and Randolph, 1993). From stage 10 to stage 13, when the medial otic placode is in close contact with the neural tube, laminin delineates each epithelial basal surface, with fine cross connections between the two layers. By stage 14, when otic and neural layers separate and mesenchymal cells invade the area, these thread‐like connections are no longer visible in the area where mesenchymal cells separate the two epithelial layers. Instead, fibronectin forms a fine meshwork surrounding the invading cells (Hilfer and Randolph, 1993). Collagen IV is uniformly present along the entire basal length of the otic epithelium and neural tube from before stage 10 to after stage 14. Upon mesenchymal invasion, little collagen IV‐immunoreactivity is found in the space surrounding the mesenchymal cells (Hilfer and Randolph, 1993). At 10 H.H. stage, heparan sulfate proteoglycan (HSP, syndecan) can begin to be detected by immunolabeling on the otic placode basal lamina, increasing markedly at 13 H.H. stage (Moro‐Balbas et al., 2000). Nevertheless, the distribution of HSP seems to be rather limited (Visconti and Hilfer, 2002). The above‐mentioned studies suggest that laminin plays a key role in the morphogenesis of the otic primordium (Visconti and Hilfer, 2002). Besides, laminin is ubiquitously present as a basal lamina component in this region of the embryo (Hilfer and Randolph, 1993). The importance of collagen IV, fibronectin and HSP during otic placode formation, has yet to be investigated. Interestingly, placodes and NC are capable to produce or induce ECM by themselves (Gans and Northcutt, 1983).

### Inner Ear Development, Cell–Cell Contact, and Extracellular Signaling Molecules

**Figure 3:**
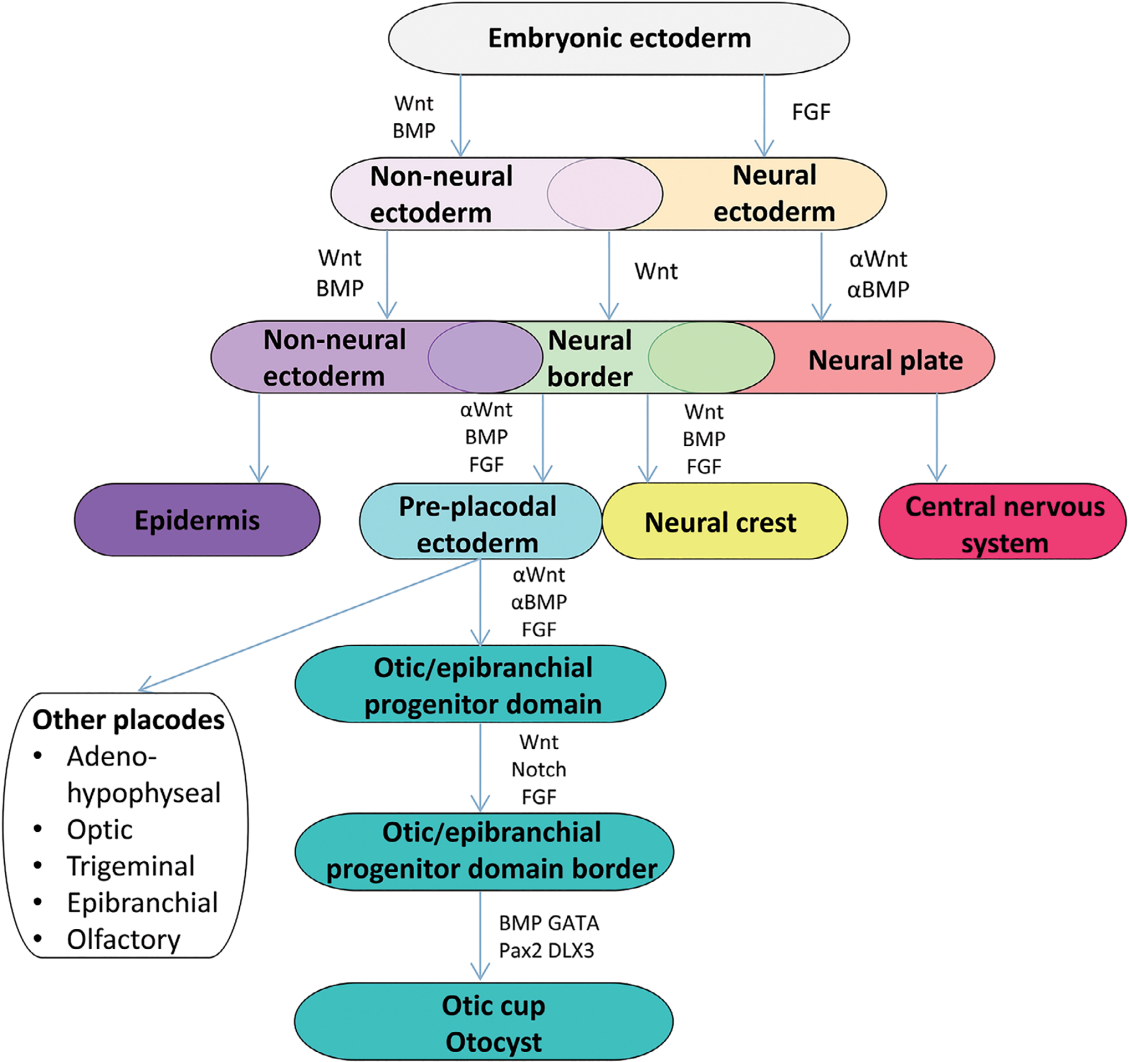
Overview of the different stages during inner ear development from gastrulation to otocyst formation. The pertinent signaling pathways involved are mentioned.

The development of specific cell types depends also on the spatiotemporal expression of extracellular signaling molecules, the signal concentration, time of exposure and combinations of different signaling molecules. Moreover, the location and numbers of the cell receptors determine the response of the cell. Notably, the identity adopted by a cell in response to a signal depends on its competence to interpret that particular signal, that is, what kind of progenitor cell it is. This, in turn, depends on what signals the cell lineage has been exposed to before (Patthey and Gunhaga, 2014). This complicated specification path holds especially true during inner ear development and it is still not known how different signaling pathways cooperate to establish the specific identity of different cell types. Around gastrulation, the ectoderm is roughly subdivided in neural and non‐neural ectoderm (Fig. 3; Streit, 2007). Interactions between these two ectodermal fields and signals from the underlying mesodermal and endodermal tissues initiate the formation of an intervening zone of ectoderm (the neural border, NB) with the potential to form the neural crest and the preplacodal ectoderm (PPE; Moody and LaMantia, 2015; Moody and Saint‐Jeannet, 2015). The cell population of the border might be dynamic at the earliest stages and intermingles with neural and epidermal precursors (in Patthey and Gunhaga, 2014, p. 13–14). In this NB zone, cells initially express a discrete set of genes, that is, “NB‐specifying” genes, which is required for the future expression of neural crest‐specific and/or PPE‐specific genes (reviewed in Meulemans and Bronner‐Fraser, 2004; Park and Saint‐Jeannet, 2010; Grocott et al., 2012). Eventually, the preplacodal region arises, consisting of a small U‐shaped band of ectoderm that encompasses the anterior neural plate. All craniofacial sensory organs (nose, lens, ear, trigeminal, and epibranchial ganglia and lateral line) arise from this PPE region (Ohyama et al., 2007; Streit, 2007).

This PPE receives inhibitory signals from the ectoderm, the neural fold and the lateral and posterior mesoderm (Basch et al., 2016). FGF, Wnt antagonists and BMP antagonists from the mesoderm which underlies the preplacodal region protect the overlying ectoderm from these inhibitory signals and allow the formation of placode precursors (Singh and Groves, 2016). Specification of cells of the preplacodal regions, including rostral placodal and neural crest cells, occurs in the region where neural‐promoting FGF signals and epidermal‐promoting BMP activity overlap and a certain balance between these two signals exist. Moreover, precursor cells from different placodes often overlap before they converge to their final destination and differentiate (Ohyama et al., 2007). Furthermore, during neural tube closure, signals that establish the anterior–posterior (A–P) axis of the embryo also enforce a regional identity on the PPE and subsequently, signals from adjacent tissues cause the PPE to separate into many discrete placodes that have distinct developmental fates (Moody and LaMantia, 2015). Loss and gain of functions of genes results in widening or reduction of the preplacodal field. Although many gene and gene functions differ between species, it is generally accepted that FGF signaling is involved in the formation of a region with Pax2‐expressing cells, which eventually can give rise to the otic placode, but which can also differentiate as epidermis (Ohyama et al., 2007). This domain, marked by early otic marker genes such as Pax2 and Pax8, has been described as the otic/epibranchial progenitor domain (OEPD), distinct from the otic placode (Whitfield, 2015). After the induction of this OEPD by FGF signals, FGF‐activity has to be reduced before Wnt signals can support otic placodal development (Freter et al., 2008; Patthey et al., 2014). The level of the canonical Wnt signaling is critical for placode‐epidermis fate decision within the preotic field. Cells which receive high levels of Wnt signaling differentiate toward the otic placode, while cells which receive little or no Wnt signaling differentiate as epidermis. It has been suggested that Wnt signaling can only influence the placode‐epidermis fate decision in the presence of species‐dependent FGF family members (Ohyama et al., 2007; Urness et al., 2010). Although it is also possible that other signaling pathways such as Notch, by regulating beta‐catenin, are important to refine the border between otic placode and epidermis (Ohyama et al., 2007). The size of the otic placode is determined mostly through Wnt signaling while several genes such as Pax2, Gata3, Dlx3, bone morphogenetic factor 4, cooperate in order to allow invagination of the otic placode to form the otic cup and subsequently, the otocyst, which is completely segregated from the ectoderm (Fritzsch and Beisel, 1998; Ohyama et al., 2007; Fritzsch et al., 2015). During invagination, the earliest axes which arise are the M–L axis and the A–P axis and shortly afterward the D–V axis. A posterior retinoic acid gradient allows for the expression of different posterior and anterior otic genes (Bok et al., 2005; Bok et al., 2011; Janesick et al., 2015). The neural tube provides a dorsalizing Wnt gradient, while these dorsalizing signals are augmented by a gradient of inhibitory Gli3R. The ventral character is established by a gradient of Shh from the notochord. During the otic placode and otic cup stages, Wnt and Fgf gradients from the neural tube help establish a medial and a lateral identity (Basch et al., 2016).

The otic vesicle is the origin of a diversity of cell types in the inner ear, such as the neurons from the VIIIth cranial ganglion, which innervate the auditory and vestibular sensory hair cells. In the otic developmental program, neurogenic/non‐neurogenic fate decision is made very early (Maier et al., 2014). Interestingly, it has been reported that the expression of Sox10 in the otic vesicle is similar to that in the neural crest, being affected when FGF or Wnt8 activity is perturbed. This supports the hypothesis that the same molecular mechanisms that induce neural crest could be important in specifying the placode region (Huisman and Rivolta, 2012)

#### In vitro *research of the generation of human inner ear progenitors (temporal dynamics)*

The principle of patterning used by Wichterle also holds true for inner ear development (Fritzsch et al., 2015). Differentiation of pluripotent stem cells into the early otic ectoderm requires suppression of the endodermal and mesodermal lineage. This can be achieved by inhibiting the TGF‐β and BMP4 and activating the FGF signaling pathways (Matsuoka et al., 2017). As mentioned in the protocol of Koehler and Hashino (2014), these signaling pathways are significant inducers of formation of the otic vesicle. Not only the concentration is of importance, the spatiotemporal pattern of many growth factors and morphogens including BMPs and FGFs are critical in the generation of inner ear progenitor cells. For example, BMP4 could only be added in a small‐time window as neuronal fate at day 5 has not the same effect as at day three. Moreover, BMP4 signaling needed to be inhibited 24–48 hr after induction to ensure a proper preplacodal ectoderm (PPE) as it happens the same in the *in vivo* mouse embryo (Kwon et al., 2010; Koehler and Hashino, 2014). FGF signaling is needed to transform the PPE toward the OEPD which contains PAX2 positive cells. In this stadium, specialization can go toward otic and epibranchial direction. The differentiation toward otic lineage depends on the duration and strength of FGF and Wnt signaling (Freter et al., 2008). Nevertheless, otic progenitors can be induced from pluripotent stem cells by activating FGF signaling with ligands involved in the generation of the placode *in vivo*, such as FGF3 and FGF10 (Chen et al., 2012). Alternatively, inner ear progenitors can be generated *in vitro*, from other stem cell populations, circumventing the above‐described procedure of stepwise addition of signaling molecules (Boddy et al., 2012). Human mesenchymal stem cells were cultured in media which previously has been used to culture human fetal auditory cells. This conditioned medium, induced the expression of otic progenitor markers, such as PAX8, PAX2, GATA3, and SOX2, while epithelial and neuronal otic precursors were morphologically distinguishable. After prolonged culture, cells coexpressed either indicators of hair cell lineage or neuronal markers such as NEUROG1, POU4F1, and NEFH.

### Inner Ear Organoids

Since the growing interest in the use of 3D‐matrices in cell culture, fast progress has been made in the generation of cerebral organoids. Lancaster et al. (2013) developed a neural organoid that contained various discrete, interdependent, brain regions. After culture of human iPSCs, using a low FGF concentration, EBs were formed. These EBs were cultured in neuronal induction medium and when neurectoderm formation started, the EBs were placed in a Matrigel™ droplet. Neuronal differentiation was initiated, using different generally used pertinent bioreactive factors. Then, the newly generated neurectoderm in the matrigel™ droplet was placed in a spinning bioreactor to provide a 3D low‐shear stress suspension culture where differentiation was completed and distinct brain regions, including hippocampus were formed after 30 days (Lancaster et al., 2013). During this period of time, the same cocktail of bioreactive factors including retinoic acid was added to the culture. Recently, the work of Lancaster was refined using a miniaturized spinning bioreactor with iPSCs, eluding the problem of large (expensive) medium volume (Qian et al., 2016). The group also developed a protocol to generate cerebral organoids in a more reproducible and quantifiable manner. Currently, this protocol, sometimes more or less modified by others, is a general procedure to achieve brain organoids.

Koehler and Hashino (2014) developed a 3D culture protocol to differentiate ESCs toward inner ear organoids that contained sensory epithelia hair cells. This protocol is more or less similar to the general principle used by Lancaster and others and largely based on the method developed by Eiraku et al. (2011) for optic cup organoids. It includes generation of an EB, initiation of neurectoderm formation and 3D culture to further develop the organoid. Later studies with the protocol of Koehler et al on inner ear organoids showed that hair cells found on the organoids developed mechanosensitivity as native hair cells in mice (Liu et al., 2016). Koehler, like many others in organoid research, used matrigel. To the best of our knowledge, studies in which organoids are generated using other matrices are yet not available.

## CONCLUSION AND PERSPECTIVE

Although it was known for years that the ECM played a crucial role during differentiation of cells, recent discoveries have revealed various cellular mechanisms that drive stem cell fate, such as mechanotransduction through focal adhesions. Increasing effort has been undertaken to create an artificial scaffold for stem cells that mimics that native ECM in the best possible ways. Promising steps were made with the use of self‐assembled peptides that makes it possible to build an artificial ECM from the ground up, that is, at amino‐acid level. Researchers already implement ECM motifs in the design of self‐assembled peptides to enhance differentiation toward a certain germ‐layer (i.e., neuronal differentiation). As ECM research in self‐assembled peptides such as RADA16 progress, more motifs could successfully be incorporated in the scaffold which makes it possible to direct stem cells toward more complex structures. Despite significant progress in research of synthetic scaffolds, these self‐assembling peptides remain a minority among matrices used in 3D culture. Control of topography and rigidity, in combination with techniques such as spinning bioreactors and microfluidic devices, made it possible to successfully culture complex structures such as cerebral organoids.

However, many limitations in culturing organoids still need to be overcome, because aspects as shape and structure, cross‐tissue communication, and patterning still do not resemble the native *in vivo* situation. As previously mentioned, when organoids grow in size, their maturation is limited due to poor oxygen supply and nutrition within deeper layers of the organoid (Chambers et al., 2013). To solve this problem, vascular endothelial growth factors (VEGFs) could be implemented into the scaffold to promote neoangiogenesis in the organoid (Richardson et al., 2001). Nevertheless, it is generally accepted that microfluidic devices can solve hyponutrition and hypoxia by integrating a controlled release of signal gradient and a constant supply of nutrients and oxygen. The disadvantage is that these systems are expensive and laborious and for that reason, they are more or less exclusively reserved for well‐equipped laboratories. Nevertheless, to study development and disease, organoids from patients own (stem) cells can be generated and explored by almost all laboratories. Pharmacological studies, the establishment of drug effects on the target‐ and perhaps also in combination with other organs, will need the use of microfluidic systems because of large numbers of tests required.

It is a key in bioengineering to combine several strategies when culturing complex higher order structures at cellular, tissue and organ level. Working with multiple cell types *in vitro* requires refinement in spatial and temporal instructions for a more coordinated differentiation. Considerable progress in regenerative medicine has been made regarding organoid and organ‐on‐chip culturing by controlling external cues, but still many challenges have to be overcome. It is of importance to realize that matrices need to be designed in such a way that spatial and temporal signaling can be controlled to guide differentiation and patterning of specific areas. Moreover, encapsulating cells within a matrix *during* polymerization of the scaffold may be beneficiary as opposed to letting cells “sink in” to their 3D environment (Nicodemus and Bryant, 2008).

The inner ear is one of the most complicated organs in the body, consisting of two related but also differently functioning parts: the cochlea and the vestibular system. To build an inner ear from human stem cells requires coupling of both the organoid of the cochlea and that of the vestibular system. Hence, a system with the capacity to support many different cell types. This may be a feasible approach as exemplified in cultures with two (and more) connected mini‐organs that were realized using microfluidic systems (Rogal et al., 2017). Developmental processes of the cochlea and/or vestibular system could be studied separately in organoid models. Subsequently, these organoids could be combined into a single microfluidic system to serve pharmacological research purposes.

It will be interesting to see how inner ear progenitor cells (Chen et al., 2012) when cultured in organoid or microfluidic culture, can be directed toward higher inner ear structures. Inner ear (progenitor) cells, whether or not (pre)differentiated in an organoid, could also be used for bioprinting purposes in which different cells and gradients of ECM can be merged. For this purpose pertinent types of ECM could be chosen, such as laminin and collagen both involved in inner ear development. To achieve the desired characteristics, for example, elasticity, these matrices could be blended with suitable biodegradable synthetic hydrogels. A certain mixture of polymers would allow changes in the composition of the matrix in time and it is conceivable that matrix alterations can be programmed, mimicking the native situation. In that perspective, self‐assembling peptide matrices have numerous advantages. They are synthesized via standard protocols and covalent incorporation of adhesive elements of choice, such as RGD or IKVAV, can be implemented on demand. They allow for 3D encapsulation of cells and frequently are injectable. Most importantly is the safety aspect: biological batch variability does not occur and these matrices are produced without animal components.

PuraMatrix™ is such a self‐assembling peptide in which the peptide sequence is assumed to have adhesive properties, which can be useful when no specific adhesive elements are needed. Both, Matrigel™ and PuraMatrix™ have the right matrix stiffness for inner ear organoids, microfluidic research, or bioprinting. Nevertheless, there are many opportunities to move further forward into this exciting field of inner ear research which has only just started.
